# A Closed-Loop Clinical Audit of Preoperative Antibiotic Prophylaxis Adherence for Surgical Procedures at Care Medical Hospital-Alrawabi in Riyadh, Saudi Arabia

**DOI:** 10.7759/cureus.115111

**Published:** 2026-08-24

**Authors:** Abdelkarim S Shaat, Joud R Hussein, Ahmad H Osman, Mahfoudh M Al Asali, Khaled A Al Mohaimeed

**Affiliations:** 1 General Surgery, Care Medical Hospital - Alrawabi, Riyadh, SAU; 2 General and Colorectal Surgery, Care Medical Hospital - Alrawabi, Riyadh, SAU

**Keywords:** antibiotic prophylaxis, clinical audit, guideline adherence, preoperative antibiotics, quality improvement, surgical site infections

## Abstract

Background

Preoperative antibiotic prophylaxis plays a vital role in preventing surgical site infections (SSIs) and supporting antimicrobial use. Although clear international guidelines exist, gaps in antibiotic selection, dosing, and timing persist in daily surgical practice. This closed-loop audit was conducted to evaluate current adherence to international prophylaxis guidelines in general surgery and assess the impact of targeted quality improvement interventions.

Methods

A retrospective clinical audit was performed at the General Surgery Department of Care Medical Hospital-Alrawabi, Saudi Arabia. A total of 130 surgical cases were evaluated, with 65 cases included in each audit cycle. The initial cycle assessed compliance with recommended antibiotic selection, appropriate weight-based dosing, and administration within 60 minutes before skin incision. After identifying key deficiencies, structured interventions were implemented, including staff education, reinforcement of guideline-based prescribing, and improved coordination between the surgical and nursing teams. A re-audit was subsequently undertaken to evaluate changes in practice, and compliance rates between cycles were compared statistically.

Results

Baseline findings demonstrated low adherence to preoperative antibiotic prophylaxis guidelines. A total of 65 patients were evaluated during the baseline audit cycle. Among patients eligible for assessment of prophylactic antibiotic administration, the correct antibiotic dose was administered in 21 (56.75%) patients, and appropriate timing within 60 minutes before skin incision was achieved in 25 (67.56%) patients. Preoperative prophylaxis was administered correctly in 15 (40.54%) patients, while overall adherence to guideline recommendations among all patients was 15 (23.07%). Following the quality improvement interventions, a second group of 65 patients was evaluated during the follow-up audit cycle. Significant improvements were observed. Correct dosing increased to 55 (96.49%) patients (*p* < 0.001), timely administration improved to 54 (94.73%) patients (*p* = 0.001), and correctly administered preoperative prophylaxis increased to 54 (94.73%) patients (*p* < 0.001). Overall guideline adherence increased significantly to 54 (83.07%) patients (*p* < 0.001). In addition, all patients in the second audit cycle received prophylactic antibiotics; cefazolin use increased substantially, and inappropriate broad-spectrum antibiotic use was eliminated. Overall, 130 patients were evaluated across the two audit cycles (65 patients in the baseline cycle and 65 patients in the follow-up cycle).

Conclusion

This audit demonstrated that structured education and coordinated system-based changes can significantly improve adherence to preoperative antibiotic prophylaxis standards. Continuous auditing and stewardship efforts are essential to sustain improvements, optimize surgical care, and enhance patient safety.

## Introduction

A surgical site infection (SSI) is a type of healthcare-associated infection (HAI). It is an infection that occurs in parts of the body where surgery takes place [1]. These infections occur within 30 days after a surgical operation (or within one year if an implant is left in place after the procedure) and affect either the incision or the deep tissue at the operation site. These infections may be superficial or deep incisional or involve organs or body spaces [2]. Beyond their clinical impact, SSIs impose a heavy financial burden on healthcare facilities, significantly prolonging hospital stay and increasing readmission rates across low- and middle- income countries' hospitals [3].

Different organisms cause infections after surgery, the most common of which include *Staphylococcus*, *Streptococcus*, and *Pseudomonas*. Pathogens can infect a surgical wound through various forms of contact, such as contact with a contaminated caregiver or surgical instrument, through germs in the air, or through germs that are already on or in the body and then spread into the wound [4].

Administering appropriate antibiotics within 60 minutes before surgical incision helps eliminate common pathogens that might cause SSIs [5].

However, adherence to international standardized guidelines [6-8] remains variable across institutions and surgical specialties. Inappropriate antibiotic choice, missed combination, delayed administration, incorrect dosing, and excessive postoperative continuation are frequently reported in the literature and may contribute not only to suboptimal infection prevention but also to the growing global threat of antimicrobial resistance [6-8]. Regular audit and feedback are essential components of antimicrobial stewardship and quality improvement initiatives [9].

This study aimed to evaluate adherence to established preoperative antibiotic prophylaxis guidelines among patients undergoing surgical procedures in the General Surgery Department at Care Medical Hospital-Alrawabi, Saudi Arabia. The evaluated components included appropriate antibiotic selection, weight-based dosing, timing of administration, and duration of prophylaxis. By identifying gaps between current practice and recommended standards, this audit sought to inform targeted interventions to improve guideline adherence and antimicrobial prescribing practices.

## Materials and methods

Study design and setting

This retrospective observational clinical audit was conducted in the General Surgery Department at Care Medical Hospital-Alrawabi, Saudi Arabia. The study was not blinded; however, during the initial audit cycle, 1-30 June 2025, healthcare personnel were not specifically informed that their individual performance was being assessed. In routine practice, surgeons were responsible for selecting and prescribing prophylactic antibiotics, while the anesthesiology team and operating room nurses were involved in their timely administration before surgical incision. Patient data were collected from medical charts and electronic medical records using a standardized checklist applied consistently to all cases. The checklist was designed to assess adherence to key prophylaxis components, including appropriate antibiotic selection, timing of administration <60 minutes before skin incision, correct dose according to weight, and allergy to antibiotics. After the initial audit cycle, targeted educational interventions were provided to the healthcare team, including discussion of the audit findings with surgical staff, orientation of newly recruited residents, focused teaching for nursing staff on antibiotic timing and documentation, and coordination with the operating room coordinator to facilitate timely patient preparation. A re-audit was then conducted from 1-30 September 2025 to evaluate improvement in compliance.

Study population

A total of 130 surgical cases were included, with 65 cases in each audit cycle. Cases were selected consecutively from all eligible surgical procedures performed during each specified audit period until the target sample size of 65 cases per cycle was reached. For patients who underwent more than one surgical procedure during the study timeframe, each operation was recorded and analyzed as an independent case.

Inclusion criteria

Patients underwent surgical procedures in the general operating suite under the General Surgery Department during the study period.

Exclusion criteria

Patients with a documented or suspected preoperative infection as the primary diagnosis, as well as those patients who received non-prophylactic antibiotic therapy within 48 hours before surgery. After applying the exclusion criteria, 130 eligible patients were included in the final analysis.

Data collection

Data were extracted retrospectively from patient charts, electronic medical records, and hospital documentation systems using a structured data collection checklist (see Appendix). Recorded variables included demographic data-specifically Medical Record Number (MRN), age, and weight category (≤120 kg vs. ≥120 kg)-as well as operative details, including date of surgery, type of procedure, and incision time. In addition, the dataset captured clinical characteristics, such as wound classification (clean, clean-contaminated, contaminated, or dirty), surgical status (elective or emergency procedure), and documented antibiotic allergies.

Audit criteria

Compliance with preoperative antibiotic prophylaxis was assessed according to the guidelines published by Stanford Health Care [6], the American Society of Health-System Pharmacists (ASHP) [7], and the Saudi Ministry of Health (MOH) guidelines [8].

Preoperative prophylaxis was considered correctly administered when all three applicable criteria were met: (1) use of the guideline-recommended prophylactic antibiotic (cefazolin), (2) administration of the appropriate weight-based dose (two grams for patients weighing ≤120 kg and three grams for patients weighing ≥120 kg), and (3) administration within 60 minutes before skin incision.

Inappropriate broad-spectrum antibiotic use was defined as selection of a broad-spectrum agent or combination regimen when a guideline-recommended narrow-spectrum prophylactic agent was indicated.

The evaluated parameters included appropriate antibiotic selection, correct timing of administration before surgical incision, appropriate weight-based dosing, intraoperative re-dosing when indicated, and appropriate duration of prophylaxis, and were aligned with established international standards for surgical infection prevention.

Data were recorded using a structured checklist developed in accordance with guideline recommendations. Each parameter was classified as compliant or non-compliant based on adherence to the specified criteria, and overall compliance was defined as adherence to all applicable guideline components for a given case, in the absence of documented contraindications such as antibiotic allergy.

Surgeons were responsible for selecting and prescribing the prophylactic antibiotic, while the anesthesiology team and operating room nurses were involved in its timely administration before surgical incision.

Quality improvement intervention

Following completion of the first audit cycle, a multifaceted quality improvement intervention was implemented during the inter-cycle period. The intervention targeted surgeons and surgical residents, operating room nurses, anesthesiology staff, and operating room coordinators. Surgeons and residents participated in weekly departmental discussions and orientation sessions reviewing the baseline audit findings and reinforcing guideline-based antibiotic selection and dosing. Nursing and anesthesiology staff received focused teaching regarding administration within 60 minutes before skin incision and appropriate documentation. In addition, weekly multidisciplinary coordination meetings involving the operating room coordinator, anesthesia, and nursing leads were conducted to identify and address delays in patient transfer and peri-operative antibiotic administration. These interventions were conducted over the four-week period between the two audit cycles and were reinforced through routine peri-operative communication.

Statistical analysis 

Statistical analyses were performed using IBM SPSS Statistics version 26 (IBM Corp., Armonk, NY, USA). Continuous variables are summarized as mean ± standard deviation (SD) and compared between audit cycles using the independent samples t-test. Categorical variables are presented as frequencies and percentages and analyzed using the chi-square test or Fisher’s exact test, as appropriate. All tests were two-tailed, and a p-value < 0.05 was considered statistically significant.

## Results

A retrospective observational study was conducted on 130 patients across two audit cycles. In the first cycle, patient ages ranged from 2.92 to 79 years, with a mean value of 36.6 (± 14.4) years. Regarding sex distribution, 45 (69.23%) patients were male, and 20 (30.77%) patients were female. In the second cycle, patient ages ranged from 0.45 to 86 years, with a mean value of 35.2 (±13.55) years. Of these, 46 (70.77%) patients were males, and 19 (29.23%) patients were females. There were no statistically significant differences in age or sex distribution between the two audit cycles. The demographics of the studied groups are shown in Table 1.

**Table 1:** Demographics of the studied groups Data are presented as mean ± standard deviation (range) for continuous variables and n (%) for categorical variables. *P*-values for age were calculated using an independent-samples t-test (t-statistic = 0.56). *P*-values for sex were calculated using the Chi-square test with Yates’ continuity correction (χ²-statistic = 0.00). *Abbreviations: SD, standard deviation; t, Student’s t-test statistic; χ², chi-square test statistic

Variable		First cycle (n = 65)	Second cycle (n = 65)	Test statistic	*P*-value
Age (years)	Mean ± SD	36.61 ± 14.36	35.24 ± 13.55	t = 0.56	0.578
	Range	2.92-79	0.45-86		
Sex	Male	45 (69.23%)	46 (70.77%)	χ² = 0.00	1.000
	Female	20 (30.77%)	19 (29.23%)		

**Figure 1:**
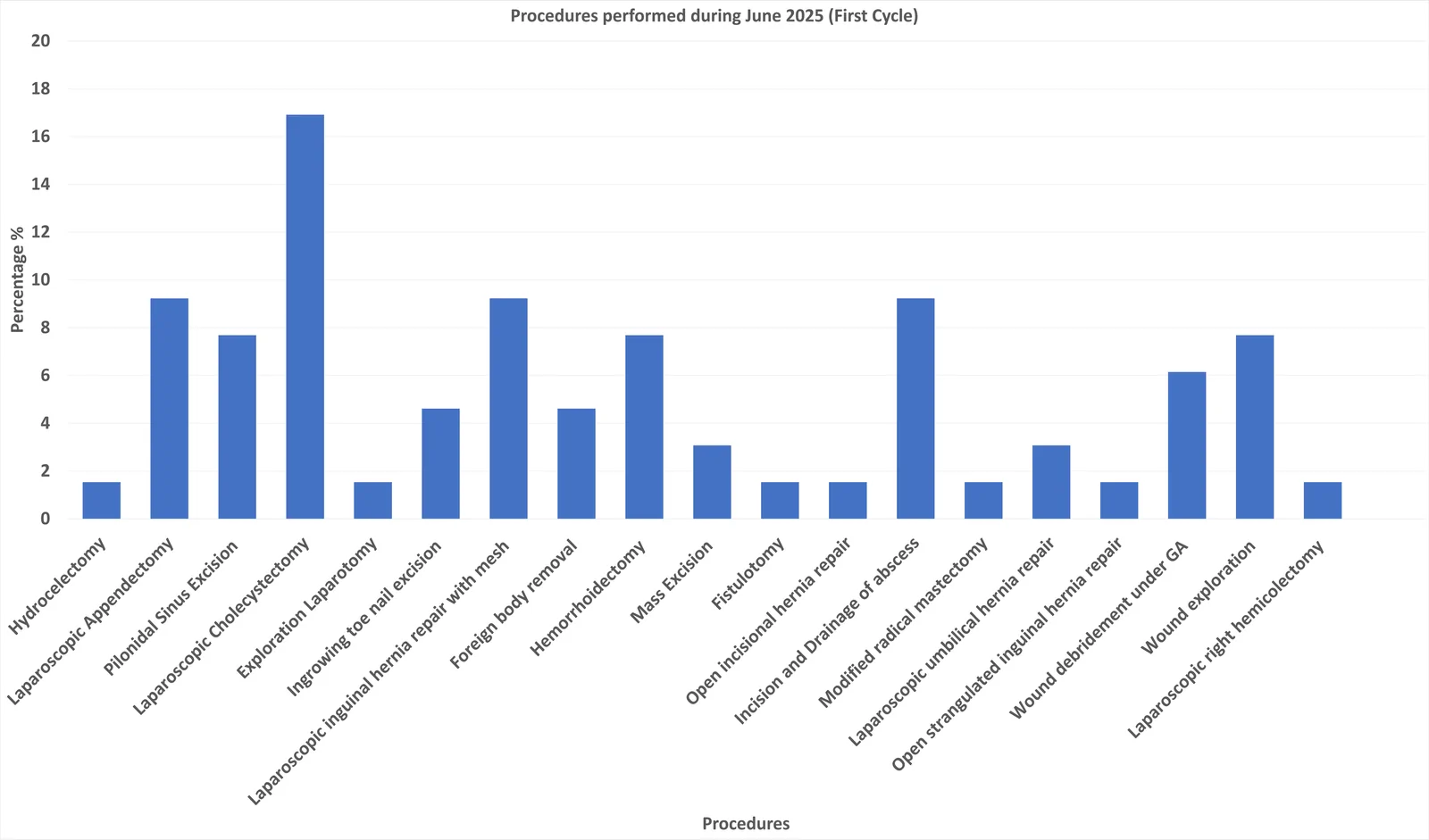
Distribution of general surgical procedures in the first cycle. Relative proportions (%) of operations evaluated during the baseline audit phase (N = 65), with laparoscopic cholecystectomy being the most frequent procedure

A total of 65 patients were included in the first audit cycle conducted in June 2025. The most frequently performed procedure was laparoscopic cholecystectomy, which was performed in 11 (16.92%) patients. This was followed by laparoscopic appendectomy, laparoscopic inguinal hernia repair with mesh, and incision and drainage of the abscess, each performed in six (9.23%) patients. Pilonidal sinus excision, hemorrhoidectomy, and wound exploration were each performed in five (7.69%) patients. Wound debridement under general anesthesia was observed in four (6.15%) patients. Ingrowing toenail excision and foreign body removal were observed in three (4.62%) patients. Two (3.08%) patients underwent mass excision and laparoscopic umbilical hernia repair. Hydrocelectomy, exploratory laparotomy, open incisional hernia repair, modified radical mastectomy, open strangulated inguinal hernia repair, fistulotomy, and laparoscopic right hemicolectomy were each observed in one (1.54%) patient. Figure 1 shows the distribution of general surgical procedures in the first cycle.

In the second audit cycle, conducted in September 2025, a total of 65 patients were also included. The most frequently performed procedure was incision and drainage of abscess, observed in 12 (18.48%) patients, followed by hemorrhoidectomy in 10 (15.38%) patients. Laparoscopic appendectomy and mass excision were each performed in five (7.69%) patients. Pilonidal sinus excision, anal fistulotomy, laparoscopic inguinal hernia repair with mesh, and open umbilical hernia repair were each performed in four (6.15%) patients. Ingrowing toenail excision and wound exploration were each recorded in three (4.62%) patients, while laser ablation of varicose veins and wound exploration with repair were performed in two (3.08%) patients.

**Figure 2:**
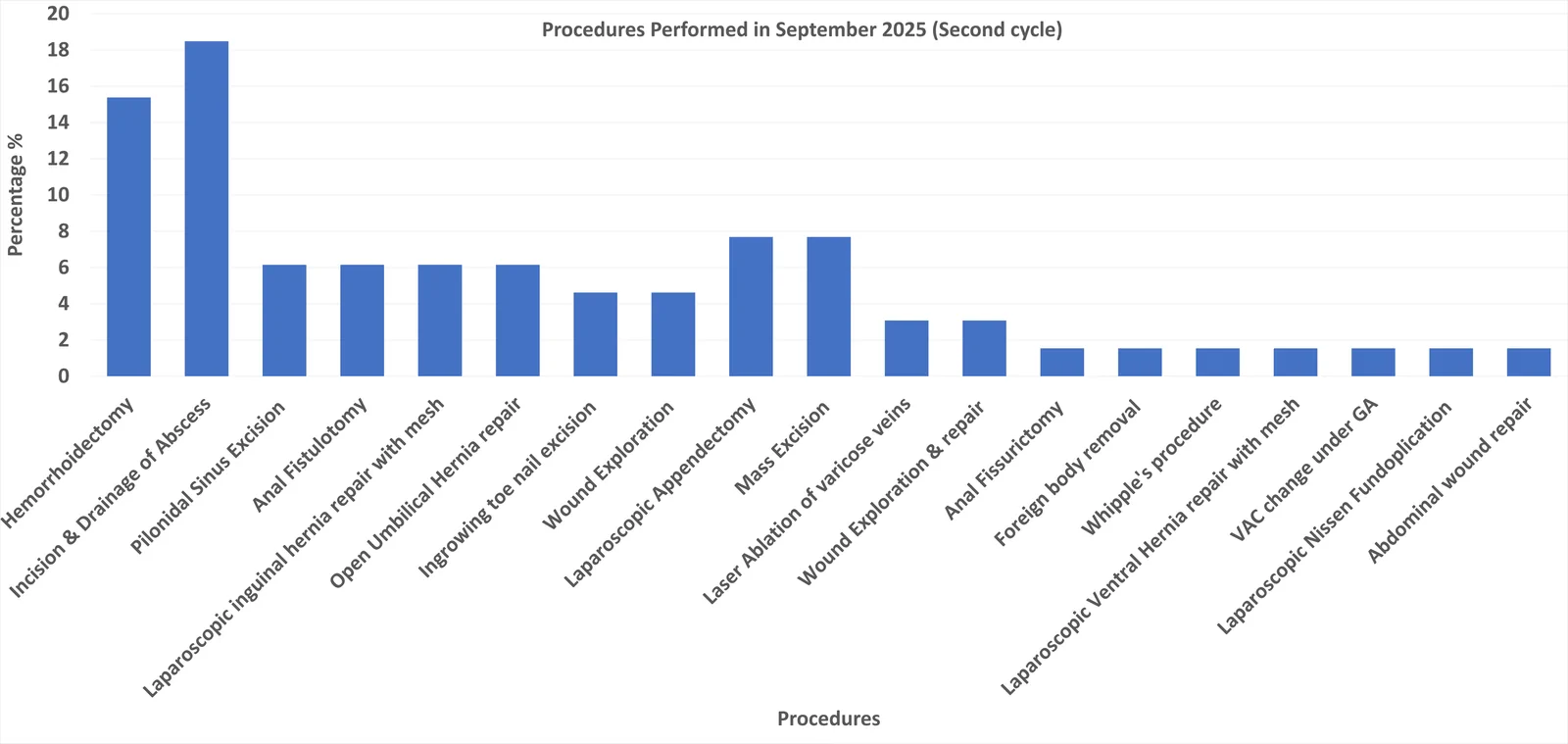
Distribution of general surgical procedures in the second cycle. Relative proportions (%) of operations evaluated during the post-intervention audit phase (N = 65), with incision and drainage of abscess and hemorrhoidectomy being the most frequent procedures.

Several procedures were performed in a single case (1.54%), including anal fissurectomy, foreign body removal, Whipple’s procedure, laparoscopic ventral hernia repair with mesh, vacuum-assisted closure (VAC) change under general anesthesia, laparoscopic Nissen fundoplication, and abdominal wound repair. Figure 2 shows the distribution of general surgical procedures in the second cycle.

**Figure 3:**
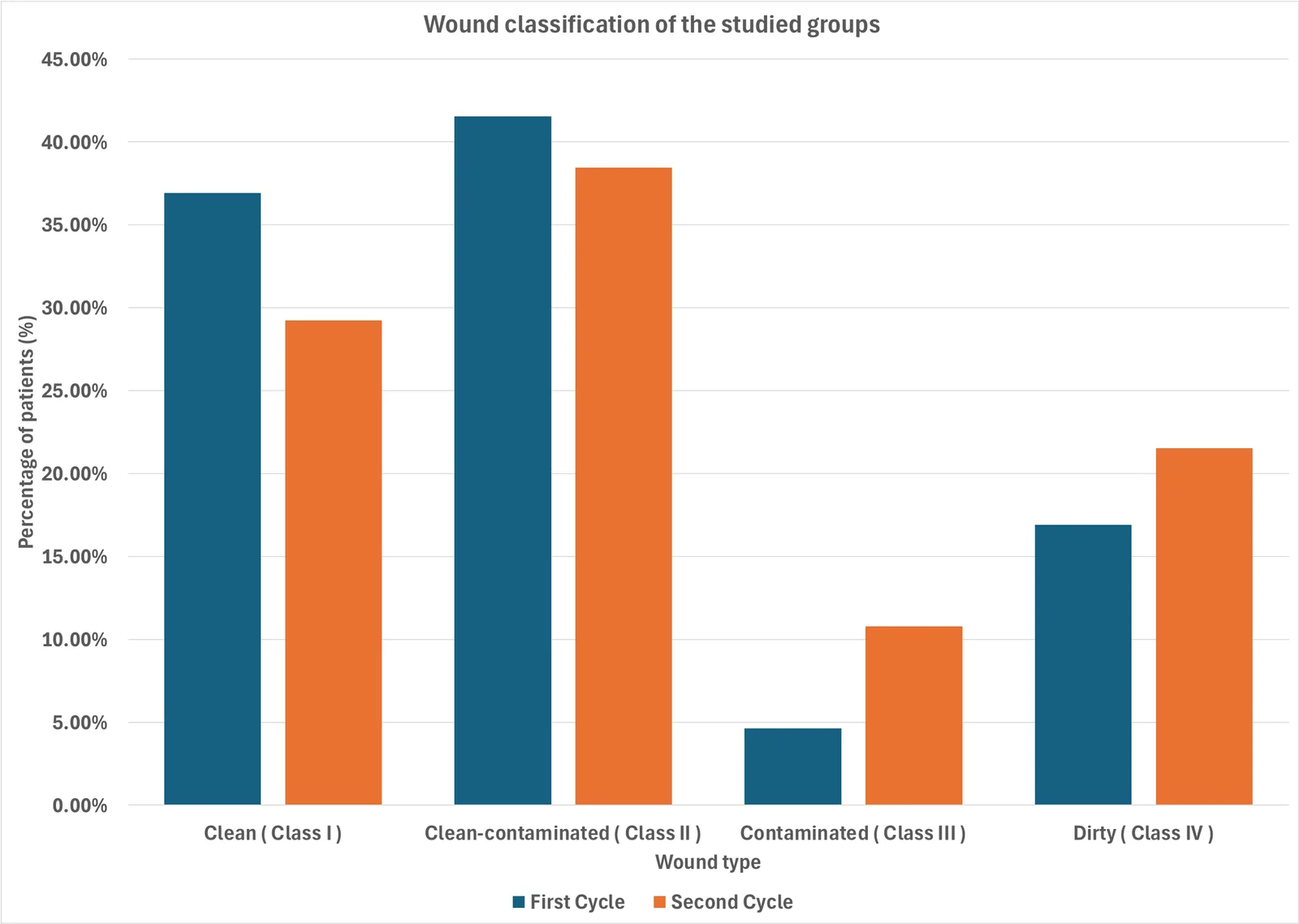
Surgical wound classifications across audit cycles. Comparison of wound categories (clean (Class I), clean-contaminated (Class II), contaminated (Class III), and dirty (Class IV)) expressed as a percentage of the total patient cohorts in the first and second audit cycles.

The distribution of wound classifications across the study groups is shown in Figure 3. In the first audit cycle (n = 65), clean wounds (Class I) accounted for 24 (36.92%) patients, whereas clean-contaminated wounds (Class II) represented the largest proportion at 27 (41.54%) patients. Dirty wounds (Class IV) comprised 11 (16.92%) patients, and contaminated wounds (Class III) were the least frequent at three (4.62%) patients. Similarly, in the second audit cycle (n = 65), clean wounds accounted for 19 (29.23%) patients, clean-contaminated wounds for 25 (38.46%) patients, dirty wounds for 14 (21.54%) patients, and contaminated wounds for seven (10.77%) patients. Although a slightly higher proportion of contaminated and dirty wounds was observed in the second cycle, statistical analysis demonstrated no significant difference between the two cycles regarding wound classification ( *p*= 0.454). These findings indicate comparable baseline wound characteristics across both audit cycles.

Table 2 presents the distribution of preoperative antibiotics administered during both audit cycles. Cefazolin was the most commonly used antibiotic in both groups, and its use increased significantly from 37 (56.92%) patients in the first-cycle group to 57 (87.69%) patients in the second-cycle group (*p* = 0.001). 

**Table 2:** Preoperative antibiotic of the studied groups Data are presented as n (%) for categorical variables. The *P*-value was calculated using the Chi-square test (χ²-statistic = 25.40, df = 8). * Indicates a statistically significant difference between groups (*P* < 0.05). Abbreviations: χ², chi-square test statistic; df, degrees of freedom

Variable		First cycle (n = 65)	Second cycle (n = 65)	Test statistic	*P*-value
Preoperative antibiotic	Cefazolin	37 (56.92%)	57 (87.69%)	χ² = 25.40	0.001*
	Meropenem and Tazocin	1 (1.54%)	0 (0%)		
	Flagyl and augmentin	1 (1.54%)	0 (0%)		
	Cefuroxime	1 (1.54%)	0 (0%)		
	Augmentin	3 (4.62%)	4 (6.15%)		
	Flagyl	4 (6.15%)	2 (3.08%)		
	Tazocin	1 (1.54%)	2 (3.08%)		
	Flagyl and Tazocin	1 (1.54%)	0 (0%)		
	Did not receive antibiotic	16 (24.62%)	0 (0%)		

The use of other antibiotics was limited in both cycles. Metronidazole (Flagyl) was administered to four (6.15%) patients during the first cycle and two (3.08%) patients during the second cycle. Broad-spectrum regimens, including meropenem with Tazocin, Flagyl with Tazocin, Flagyl with augmentin, and cefuroxime, were each used in one (1.54%) patient in the first cycle and were not used in the second cycle.

Augmentin use was comparable between the first and second cycles, with three (4.62%) patients receiving augmentin in the first cycle and four (6.15%) patients in the second cycle. Similarly, Tazocin was administered to one (1.54%) patient in the first cycle and two (3.08%) patients in the second cycle.

Notably, 16 (24.62%) patients in the first-cycle group did not receive preoperative antibiotics, whereas all patients in the second-cycle group received antibiotic prophylaxis.

There was a marked improvement in adherence to preoperative antibiotic prophylaxis standards in the second audit cycle compared with the first cycle (Table 3). Among patients who received Cefazolin prophylaxis, administration of the correct antibiotic dose increased significantly from 21 (56.75%) patients in the first cycle to 55 (96.49%) patients in the second cycle (*p* < 0.001). Likewise, administration within the recommended timeframe of less than 60 minutes before skin incision improved significantly from 25 (67.56%) patients to 54 (94.73%) patients (*p* = 0.001).

**Table 3:** Comparison of prophylactic guideline adherence between audit cycles. Data are presented as n (%) for categorical variables. *P*-values were calculated using two-tailed Pearson Chi-Square tests. A *P*-value < 0.05 is considered statistically significant. Statistical test: Values presented in the "Test statistic (χ²)" column represent Pearson's chi-square test values. *Preoperative prophylaxis was defined as correctly administered when a patient received the recommended prophylactic antibiotic, appropriate weight-based dose and the drug was administered within 60 minutes prior to skin incision. Administration-related indicators (correct dose, timing, and correctly administered prophylaxis) were calculated among patients who received the recommended prophylactic antibiotic (n = 37 and n = 57), whereas overall guideline adherence was calculated using all patients (n = 65 per cycle). Abbreviations: χ², chi-square test statistic; N/A indicates that statistical comparison could not be performed because no antibiotic allergies were documented in either group.

Variant	First cycle (n = 37)	Second cycle (n = 57)	Test statistic (χ²)	*P*-value
Correct dose	21 (56.75%)	55 (96.49%)	χ² = 22.89	< 0.001
Allergy to antibiotics	0 (0%)	0 (0%)	N/A	N/A
Timing before skin incision <60 minutes	25 (67.56%)	54 (94.73%)	χ² = 10.26	0.001
Preoperative prophylaxis given correctly*	15 (40.54%)	54 (94.73%)	χ² = 33.77	< 0.001
Overall guideline adherence among all 65 patients	15 (23.07%)	54 (83.07%)	χ² = 46.98	< 0.001

Furthermore, preoperative prophylaxis was administered correctly in 15 (40.54%) patients during the first cycle compared with 54 (94.73%) patients during the second cycle (*p* < 0.001). When overall adherence was assessed across all 65 patients in each audit cycle, compliance with the established antibiotic prophylaxis guidelines increased significantly from 15 (23.07%) patients in the first cycle to 54 (83.07%) patients in the second cycle (*p* < 0.001).

Antibiotic allergy status was retrospectively extracted from the electronic medical record; no documented antibiotic allergies or intra-operative re-dosing were identified in both audit cycles.

These findings reflect a significant enhancement in preoperative antibiotic stewardship and adherence to guideline-based practices following implementation of the second audit cycle.

## Discussion

This closed-loop audit evaluated adherence to preoperative antibiotic prophylaxis guidelines [6-8] in general surgical procedures and demonstrated a statistically significant improvement in compliance following targeted quality improvement interventions. The findings support the effectiveness of structured audit feedback and focused educational strategies in achieving measurable and clinically meaningful improvements in prescribing practices [10].

In the first audit cycle, overall adherence to recommended prophylactic standards was low, with only 15 (23.07%) patients meeting all guideline criteria. In addition, 16 (24.62%) patients did not receive any preoperative antibiotic prophylaxis, representing a substantial deviation from established international recommendations [6-8]. Among patients who received Cefazolin prophylaxis, the correct weight-based dose was administered in only 21 (56.75%) cases, while timely administration within 60 minutes before skin incision was achieved in 25 (67.56%) cases. These findings highlight significant gaps in preoperative antimicrobial practice and underscore the need for structured stewardship interventions.

Antibiotic selection patterns in the first cycle further reflect inconsistent adherence to guideline-recommended agents [6-8]. Although cefazolin was administered in 37 (56.92%) cases, broad-spectrum antibiotics such as meropenem and piperacillin-tazobactam (Tazocin) were also utilized despite their limited role in routine surgical prophylaxis [11]. Such prescribing patterns may contribute to the development of antimicrobial resistance and are inconsistent with established prophylactic principles [12], which emphasize the use of narrow-spectrum agents whenever appropriate.

Following the implementation of corrective measures, including reinforcement of guideline recommendations [6-8], educational sessions, and enhanced multidisciplinary coordination, a marked improvement was observed in the second audit cycle. The improvement followed a multifaceted intervention rather than a single educational session. The intervention consisted of repeated weekly discussions with surgeons and residents to review baseline audit findings and reinforce guideline-based antibiotic selection and dosing, orientation of newly onboarded residents, focused education of nursing and anesthesiology staff regarding prophylaxis timing and documentation, and weekly multidisciplinary coordination with operating room staff to address delays in patient preparation and antibiotic administration [13]. These activities were conducted throughout the inter-cycle period and were reinforced through routine perioperative communication before the re-audit. The intervention therefore targeted both knowledge-based gaps and system-level factors contributing to delayed or inappropriate prophylaxis.

Overall adherence to prophylactic guidelines increased significantly from 15 (23.07%) patients to 54 (83.07%) patients (*p* < 0.001), representing a substantial improvement compared with baseline. Correct weight-based dosing among patients receiving Cefazolin prophylaxis improved from 21 (56.75%) patients to 55 (96.49%) patients (*p* < 0.001). Likewise, timely administration within 60 minutes before skin incision improved significantly from 25 (67.56%) patients to 54 (94.73%) patients (*p* = 0.001), indicating enhanced coordination between the surgical and nursing teams.

Importantly, the omission of prophylaxis was eliminated in the re-audit cycle, with 100% of the patients receiving preoperative antibiotics. This represents a significant improvement in patient safety and adherence to standard practice. Furthermore, Cefazolin utilization increased significantly from 37 (56.92%) to 57 (87.69%) patients, reflecting improved alignment with international guideline recommendations [6-8]. Concurrently, the use of inappropriate broad-spectrum antibiotics was eliminated in the second cycle, demonstrating strengthened antimicrobial stewardship and rational prescribing practices.

The statistically significant p-values across the major compliance indicators suggest that the observed improvements were unlikely to have occurred by chance. In particular, the increase in overall guideline adherence from 15 (23.07%) to 54 (83.07%) patients demonstrates the effectiveness of the closed-loop audit model in promoting sustainable practice modification [10]. The degree of improvement observed in this audit is comparable and highlights the responsiveness of surgical teams to structured performance feedback [14,15].

Although this audit did not directly measure SSI rates, improved adherence to appropriate antibiotic selection, dosing, and timing has been consistently associated in the literature with reduced postoperative infection risk [16]. Therefore, the improvements observed in this study are likely to contribute to enhanced patient safety, optimized antimicrobial utilization, and a reduction in overall healthcare burden.

Despite its strengths, this audit had certain limitations. This study was conducted at a single institution with a moderate sample size, which may limit the generalizability of the findings.

In summary, this closed-loop audit demonstrated that targeted educational and system-based interventions significantly improved adherence to the preoperative antibiotic prophylaxis guidelines [6-8]. These findings reinforce the importance of continuous quality improvement efforts to optimize surgical care, strengthen antimicrobial stewardship, and promote patient safety.

## Conclusions

This closed-loop clinical audit demonstrated a clinically meaningful improvement in adherence to preoperative antibiotic prophylaxis guidelines following targeted quality improvement interventions. Baseline evaluations revealed substantial gaps in antibiotic selection, dosing accuracy, and timing of administration.

Following the implementation of the improvement measures, compliance was reinforced through ongoing communication with the surgical and operating room teams, including discussions of audit findings, staff orientation, and reinforcement of appropriate antibiotic timing and documentation practices. These measures helped support the improvement observed in the re-audit and may contribute to sustaining compliance over time. To maintain these gains and further optimize patient care, the hospital will continue regular monitoring of perioperative antibiotic prophylaxis compliance through periodic audits and reinforce adherence to established guidelines through ongoing staff education. Incorporating antibiotic timing and documentation into routine surgical safety practices will integrate these principles into daily surgical pathways, further supporting long-term compliance.

## Figures and Tables

**Figure 4:**
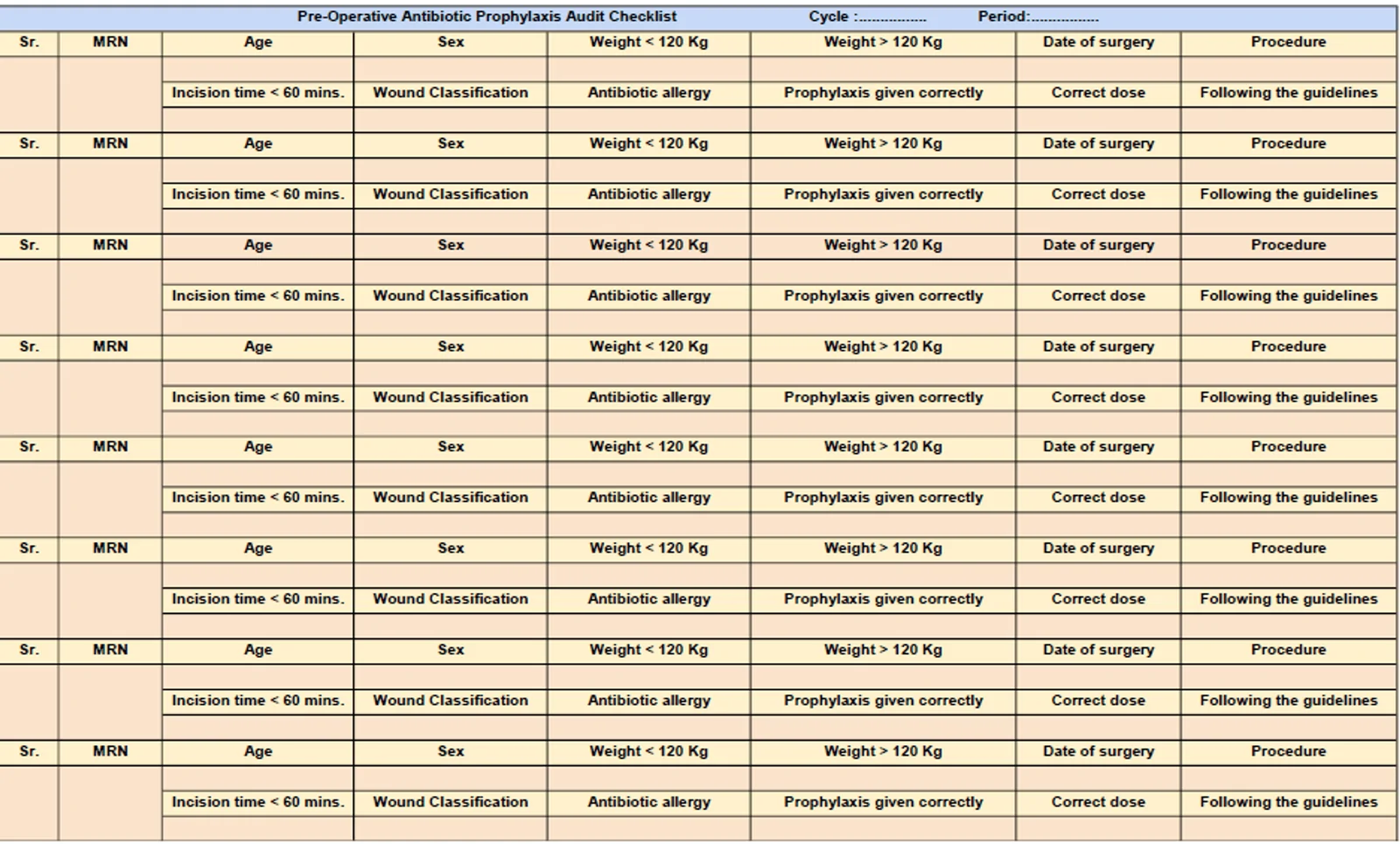
Data collection checklist used for study extraction and analysis Data collection audit sheet used to record patient demographics, preoperative antibiotic selection, weight-based dosing, incision timing, and guideline compliance during audit cycles. Abbreviations: MRN, Medical Record Number; Sr., serial number; Kg, kilograms; mins., minutes
